# Evidence synthesis as the key to more coherent and efficient research

**DOI:** 10.1186/1471-2288-9-29

**Published:** 2009-04-30

**Authors:** Alexander J Sutton, Nicola J Cooper, David R Jones

**Affiliations:** 1 Department of Health Sciences, University of Leicester, Leicester, UK

## Abstract

**Background:**

Systematic review and meta-analysis currently underpin much of evidence-based medicine. Such methodologies bring order to *previous *research, but *future *research planning remains relatively incoherent and inefficient.

**Methods:**

To outline a framework for evaluation of health interventions, aimed at increasing coherence and efficiency through i) making better use of information contained within the existing evidence-base when designing future studies; and ii) maximising the information available and thus potentially reducing the need for future studies.

**Results:**

The framework presented insists that an up-to-date meta-analysis of existing randomised controlled trials (RCTs) should always be considered before future trials are conducted. Such a meta-analysis should inform critical design issues such as sample size determination. The contexts in which the use of individual patient data meta-analysis and mixed treatment comparisons modelling may be beneficial before further RCTs are conducted are considered. Consideration should also be given to how any newly planned RCTs would contribute to the totality of evidence through its incorporation into an updated meta-analysis. We illustrate how new RCTs can have very low power to change inferences of an existing meta-analysis, particularly when between study heterogeneity is taken into consideration.

**Conclusion:**

While the collation of existing evidence as the basis for clinical practice is now routine, a more coherent and efficient approach to planning future RCTs to strengthen the evidence base needs to be developed. The framework presented is a proposal for how this situation can be improved.

## Background

Over the last two decades we have experienced the evidence-based medicine (EBM) revolution [1] in how interventions are evaluated and administered. Central to this initiative is the use of systematic review and meta-analysis of randomised controlled trials (RCTs), since they provide the highest level of evidence regarding effectiveness of interventions. This has led to an increasing reliance on the use of meta-analysis to inform clinical decision-making at both the policy and individual level. Additionally, it is often stated that one of the outputs of a systematic review is to identify "gaps" in the current evidence base, and this is made explicit in the aims of the Cochrane Collaboration [2]. To this end, a systematic review should inform future research and, indeed, the QUOROM (recently renamed PRISMA) statement checklist [3] includes the item "suggest a future research agenda". Not only is this desirable, but doing otherwise is incoherent and will lead to inefficiency through the design of sub-optimal RCTs in the future [4]. However, recommendations currently found in systematic reviews regarding research needs, although useful, [5] could be made more informative and explicit.

Further, presently, the vast majority of meta-analyses are produced as observational by-products of the existing literature; little or no consideration of the overarching (meta-) analysis is made at the design stage of the individual component studies that eventually make up the meta-analysis. This is despite the fact that in many instances *the updated meta-analysis will be of central importance and more influential than the results of the new studies on their own *(as implied by the position of meta-analyses at the top of hierarchies of types of evidence[6]). If we accept this point of view, then it is coherent to design and power a new trial based on the predicted results of the updated synthesis of the existing evidence, rather than powering the new trial on an isolated analysis [7].

To address this incoherence, we propose a cyclic framework for evaluation of interventions, incorporating emerging methodologies, aimed at increasing coherence and efficiency through i) making better use of information contained within the existing evidence-base when designing future studies; and ii) maximising the information so gained and thus potentially reducing the need for future RCTs, and the costs and delays they entail.

**Figure 1:**
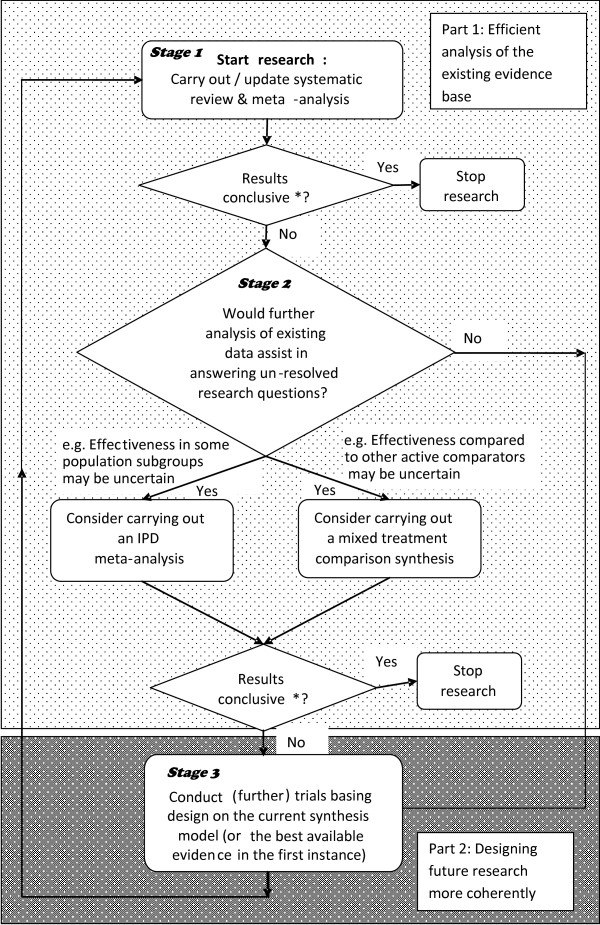
**Flow-chart for proposed cyclic, coherent and efficient research synthesis/research design strategy for answering questions of clinical importance**. * Ideally based on a clinically-centred criteria such as limits of equivalence (rather than statistical significance).

If implemented, we believe this would go some way to ensuring future research is more evidence-based. As well as reducing the economic cost of gaining further information (which is what we imply by efficiency here) we believe such methods also potentially have benefits from an ethical perspective by maximising the information gained for each new patient randomised. Figure 1 summarises the whole cyclic framework; the exposition that follows fleshes out the three stages contained within the two distinct parts to the framework outlined in the Figure.

## Methods

### Part 1: Analysis of the existing evidence-base

Stage 1: Before any new study is designed, it is important that an up-to-date systematic review and meta-analysis is identified or carried out, as those which are published potentially go out of date quickly [8].

Even if this does not answer the clinically important questions of interest, it may be possible to answer them through further analysis of the existing evidence-base (Stage 2). Several evidence synthesis models are described together with their potential advantages over standard meta-analysis for answering increasingly important clinical questions. These methods are included in the flow-chart (Figure 1), indicating how they fit into the cyclic approach to research design and analysis, as such models can be used as the basis of designing of future studies in the same way as meta-analysis can be (see Stage 3 in Part 2 below).

#### Detailed analysis of patient subgroups: obtaining and analysing Individual Patient Data (IPD)

If there is still uncertainty regarding the effectiveness of an intervention in certain population subgroups (an increasingly important issue as we move towards individualising treatment regimes) it may be worthwhile obtaining and analysing individual patient data (IPD) [9] since this would allow a much more powerful exploration of *patient characteristic by treatment interactions *[10]. Although such analyses are more costly than meta-analyses based on summary statistics, they may still be considered "good value" compared to conducting future trials. Further, "half-way house" alternatives to obtaining the IPD, such as requesting summary statistics on specific subgroups of patients, may provide a useful compromise with respect to cost and power [11]. In the past, meta-analysis of IPD were sometimes hampered by the lack of availability of data from a proportion of the relevant studies, but methods are being developed [12] to combine IPD and summary data allowing most efficient use of the available data in such situations. Such methods have been used to explore the influence of socio-economic variables on home safety practices [13] including the safe storage of matches or lighters considered in the results section. However, as documented elsewhere, [14] methods for identifying subgroup effects can be abused and results over-interpreted and measures should be taken to avoid this.

#### Synthesis of all competing interventions: Mixed treatment comparisons

An important development in evidence synthesis methodology over recent years is mixed treatment comparison (MTC) modelling [15-18]. Such an approach allows evidence networks in which trials making different comparisons to be synthesised together allowing a coherent picture of the effectiveness of all treatment options to be created. This allows i) the estimation of comparisons for which there is no direct randomised evidence – enabling us to answer such clinically relevant questions as the probability any one treatment is superior to the rest; ii) an increase in precision of treatment effects where there are direct estimates by including the indirect comparisons; and iii) an exploration of the consistency of the whole evidence-base for a particular condition. The potential to amalgamate existing meta-analyses, [19] as well as create new syntheses from the ground-up is considerable.

#### Utilising other evidence synthesis models

There is also scope for using other evidence synthesis structures to answer questions of clinical importance, and these are reviewed in detail elsewhere [20]. For example, the use of surrogate endpoints has received much attention recently [21] and synthesis models have been described which combine information on both intermediate and clinical outcomes using a "chain of evidence" approach [22] making efficient use of multiple sources of information. Further models, utilising economic data are considered in the discussion.

### Part 2 (including Stage 3): Designing future research more efficiently

#### Role of systematic review and meta-analysis in the design of future research

Although, in some contexts, it is becoming obligatory to consider the current evidence-base when designing new studies (e.g., in the UK applicants to the Medical Research Council/National Institute for Health Research for clinical trial support are prompted to provide evidence from systematic reviews of the *need *for their proposed trial). Considering how the results of newly completed studies update the evidence-base [23] has become a requirement when publishing RCTs in some journals including JAMA and The Lancet [24] and encouraged by others. However, there is still a rift in the cyclic process of designing new studies using current knowledge, synthesising the results of such studies with the existing research and then designing further studies informed by these results to answer the questions of clinical importance (Figure 1). Evidence exists to suggest that such a cyclic process is not adhered to routinely. For example, Cooper et al [25]. found that only 8 out of 24 trialists (33%) consulted the Cochrane (or other) systematic review (in existence by 1996), which their new RCT would ultimately update (in either 2002 or 2003), when designing their RCT. One of the reasons new research may not be designed as coherently as it could have been, using the existing evidence-base, is that there is remarkably little written on how to do this, at least quantitatively. While it is already widely recognised that previous research may assist in defining such important details as i) outcome definitions; ii) hypotheses under test, as well as making sure pitfalls of previous studies are avoided, [4] less consideration has previously been given to *quantitative *design issues, such as the sample size of a future RCT.

As noted in the introduction, little consideration of the overarching (meta-) analysis is made at the design stage of the individual component studies that eventually make up the synthesis, despite the fact that the updated meta-analysis may be more influential than the results of the new studies on their own. Therefore, it is coherent to design and power a new trial based on the predicted results of the updated meta-analysis (or related synthesis model) including the new trial (Stage 3 in Figure 1) [7].

Although effectiveness of an intervention is still often defined in terms of its statistical significance, it is also well appreciated that *p*-values are limited in their usefulness [26]. A particular limitation is that interventions that are statistically significant may not be clinically important. In such circumstances, data collection should stop when it has been ascertained that, even if an effect exists, it is unlikely to be clinically important; this will be prior to the null hypothesis (of no treatment effect) being rejected. If this does not happen then much resource could be wasted 'chasing' sub-clinical or non-existent effects in future research.

An alternative to statistical significance, which addresses this issue, is to define limits of equivalence using pre-specified criteria to define effect sizes for which a new treatment is clinically superior to the existing one [7]. If this were done then design decisions would be based around the estimate of effect and associated uncertainty. For example, data collection could stop once it was 95% certain that the effect size was within or outside the limits of equivalence. (N.B. this notion is closely related to how equivalence trials are currently designed [27]). Despite obvious challenges in definitively choosing values for the limits of equivalence, the authors are in support of the use of this type of criterion as an alternative to arbitrary cut-offs on the p-value scale for decision making.

In the results section we consider alternatives to the traditional sample size calculation which use the current evidence-base as their basis, including approaches that consider how the new trial results will impact on the totality of the evidence-base and use criteria other than statistical significance.

There is still the need to design the first RCT, when no previous ones have been conducted. In such cases the use of traditional sample size calculations may be appropriate. For example, designing a trial with favourable power to detect the smallest difference that would be clinically worthwhile using evidence from related studies (e.g. drugs in the same class etc.), ideally taking the uncertainty in the estimation into account [28]. However, there may be advantages in formalising this procedure through further methodological development. This could help avoid sample size calculations being based on inflated control group rates and excessively large treatment effects. There is even evidence to suggest sample size calculations are sometimes "reverse engineered", with the treatment difference assumed in the power calculation being derived from the given economic/feasibility restrictions on the researchers [29]. For whatever reason, when this occurs, the RCT is likely to be underpowered and this is not an efficient (even ethical) use of resources.

As with more ad hoc updating of meta-analyses, the threat of spurious results being obtained, due to multiple analyses being conducted as data accumulates within the framework, remains a possibility. Previous authors have considered this issue for updating of meta-analyses assuming fixed effect [30] and heterogeneous data accumulation, [31] (Higgins JPT, Whitehead A, Simmonds M: Sequential methods for random-effects meta-analyses, submitted) adapting formal monitoring procedures initially developed for single RCTs. We would fully support the use of such approaches in conjunction with the framework presented (as we would the adaptation of methods for adaptive trial design used to maintain type-1 error rates [32]).

## Results

### Estimating the power of a new trial, based on statistical significance, using the results of an existing meta-analysis

**Figure 2:**
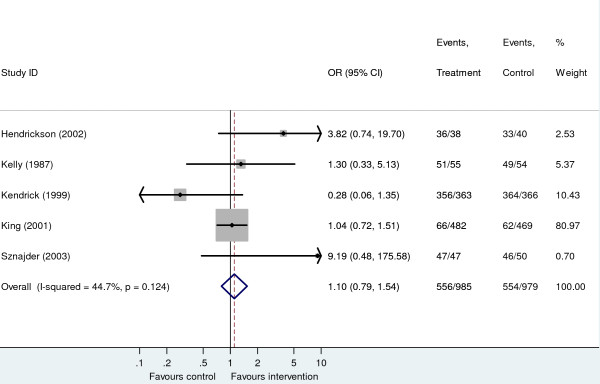
**Fixed effect meta-analysis from home safety education and provision of safety equipment for injury prevention systematic review for the outcome storage of matches or lighters out of reach of children**.

Figure 2 presents an existing meta-analysis, taken from a recent Cochrane review [13], of the success of households at storing matches or lighters out of the reach of children following education and/or provision of safety equipment. A fixed effect analysis produces an odds ratio of 1.10 (95% CI 0.79 to 1.54). Hence, although the point estimate suggests a potential modest benefit of the intervention, the result is non-significant at the 5% significance level (p = 0.56) and the confidence interval includes one, suggesting a need to collect more evidence before firm conclusions on the effectiveness of the intervention can be made. Alternative approaches to estimating the sample size for a new trial are outlined below.

### Powering a new trial in isolation, based on statistical significance, using the meta-analysis

A new trial could be designed with the expected treatment effect being equal to the pooled estimate of effect from the meta-analysis. Although traditionally sample size calculations are often based on the smallest clinically worthwhile effect size, the same mathematical approach can be applied using the meta-analysis point estimate instead since this can be considered the *expected *treatment effect. Specifically, Type I and II error rates can be specified in the usual way and sample sizes can be derived using standard formulae [33]. For the example meta-analysis, if we wish to have 80% power to detect an odds ratio of 1.10 at the 5% level in a new trial, assuming the baseline event rate in the new trial equal to the average observed in the meta-analysis, (N.B. this is also assumed in the alternative approaches considered below) approximately 1200 subjects per trial arm would be required. Clearly, in this and the examples which follow, improved, context-specific, estimates of the control group event rate may be available, for example, through the use of relevant observational data, ideally, measured in the proposed trial population [29]. If so, we would encourage the use of such estimates as an alternative to averaged estimates.

### Powering a new trial to update the existing meta-analysis based on statistical significance

As argued in the main text, it is more coherent to power a trial with respect to the impact it has on a potential future updated meta-analysis if this will be used to make decisions. Recently, we have described a method to do this [7] which uses simulation methods and takes into account the uncertainty in the pooled meta-analysis estimate. For the example meta-analysis, the sample size for a new trial to provide 80% power to yield a fixed effect *meta-analysis *(i.e. ignoring between study heterogeneity) with a statistically significant result at the 5% significance level would require 14,000 subjects in each arm. This number is so large, for this example, because of the small effect size estimated from the meta-analysis (i.e. if an intervention effect does exist, it is likely to be small) and the large uncertainty in its estimation. The sample size derived by this method can be larger or smaller than that calculated using standard methods depending on the treatment effect and corresponding uncertainty obtained from the meta-analysis of the existing studies. The greater the amount of existing evidence, the greater the 'inertia' to change by new evidence in the pooled effect.

Although the above calculation ignored heterogeneity between study results in the meta-analysis, in fact, moderate heterogeneity is present (suggested by an *I*^2 ^value [34] of 44.7%) and this should be taken into consideration when designing future studies. Ideally, it is highly desirable to try and explain such heterogeneity by including study-level covariates in the meta-analysis model [35] or conducting subgroup analyses (or obtaining individual patient data (IPD) as discussed in Part 2). If study/patient characteristics are identified which appear to influence outcome, then obviously these factors need to be considered when designing a new study. However, such endeavours, based on summary level data are often limited, as they are restricted by the small number of studies typically included in a meta-analysis [10]. Residual heterogeneity can be accounted for using a random effects model. One of the implications of including a random effect in a meta-analysis model is that it acts as a limiter on the magnitude of the influence any individual study has on the analysis. This implies that when heterogeneity is present, even a very large new study may have very little (even zero – see below) power to change statistical inferences in a meta-analysis (!) since its allocated weight will be relatively small, and inevitably under-powered in the meta-analysis framework (even though it could have considerable power when analysed on its own). This is indeed the case for the storage of matches example; *given the degree of heterogeneity, no single further study, irrespective of its size, has any power to produce a significant pooled result at the 5% level when included in the updated meta-analysis*.

Famous examples of this phenomenon exist in the literature. For example, in a random effects meta-analysis of trials of magnesium trials in myocardial infarction, [36] the mega-trial ISIS-4 includes 93% of the total number of patients randomised across all trials but receives only 17% of the weight in a random-effects meta-analysis.

An implication of this is that multiple small new studies can, in aggregate, be weighted more than a single large study containing the same number of subjects as the smaller studies in total in an updated meta-analysis; this has important implications for design of future studies. A heuristic explanation for this finding is that multiple estimates can better estimate the *distribution *of effects assumed under a random effects model. We do not believe this point is widely appreciated as it should be and it is seldom taken into consideration when new studies are designed.

### Powering a new study to update the existing meta-analysis based on limits of equivalence

In order to use this approach it is necessary to specify effect sizes which are too small to be important, that is, in this context, effect sizes for which it would not be considered worthwhile to offer education/provision of safety equipment to prevent thermal injuries. Using simulation, estimation of power for such criteria is straightforward as illustrated elsewhere [7]. For example, if it were considered that odds ratios of less than 1.5 represent non-worthwhile effect sizes, approximately 850 subjects in each arm of a new trial would be required to have 80% power to *rule out *effect sizes greater than this with a type 1 error of 5% under a random effects model. (I.e. this further trial would be done with a view to definitively establishing the effectiveness of the intervention is not of a worthwhile magnitude.)

## Discussion

In this paper we have presented a coherent and efficient framework for research design underpinned by evidence synthesis methodologies. We have kept technical details to a minimum, although these can be found in the cited articles. While the context considered throughout is the design of RCTs to evaluate interventions, the framework and methodologies are relevant, and readily modifiable, to other research areas such as epidemiology.

There have been previous efforts to designing multiple studies in a coherent manner. For example when carrying out multiple studies simultaneously, prospective meta-analyses [37,38] have been used to design a collection of studies with the prospect of an eventual meta-analysis in mind, so that important elements of study design complement each other across studies [39]. While our sequential approach complements this simultaneous design approach, it will be applicable in a broader range of contexts. Others have also considered the use of (fully) Bayesian methods which utilise previous evidence to derive prior distributions to inform sample size calculations for new studies, [40] but the updating of evidence is via Bayes Therom and not via meta-analysis and hence does not model heterogeneity in the same way.

For the framework and sample size calculations of future trials to be valid, the meta-analysis it includes also needs to be valid. Therefore, the usual threats to the validity of a meta-analysis, including the threat of publication bias and biases induced by the inclusion of sub-optimally conducted trials, need to be considered and ideally addressed using methods which are continually evolving [41,42]. A particular threat to the sequential framework is time-lag bias, with interesting results published more quickly than the rest. Recursive cumulative meta-analysis, or meta-regression, could be used to check for this and for other reasons why effect sizes may systematically change over time [43].

Returning to the magnesium for myocardial infarction meta-analysis, in this example, the mega-trial ISIS-4 produced results which were inconsistent with the previous trials by suggesting magnesium was ineffective. This is an example where a random effect meta-analysis model would not appear to be a suitable model for this data and a more measured analysis would consider both study sample size and patient baseline risk as important covariates which need to be taken into consideration [36]. This highlights the challenge of needing to choose an appropriate model that not only combines the previous evidence, but the future evidence also. Further, generally, too little thought has gone into interpretation when (unexplainable) heterogeneity exists in random-effects meta-analysis; although this is starting to be addressed [44]. When such heterogeneity exists it can seriously diminish the impact future research will have (e.g. even the huge ISIS-4 trial had minimal impact on the existing evidence base); although this is an issue with random effect models rather than our framework *per se*. This can be viewed as undesirable, and we are certainly not opposed to the exploration of approaches to dealing with heterogeneous data that do not involve random effects.

We acknowledge that before widespread adoption of the framework can take place careful development of case studies is desirable to consider specific implications in more detail (e.g. such as the role of confirmatory trials) and further refine the methods, especially when using evidence structures other than standard meta-analysis to inform future research. In particular, the evaluation of such methods in the pharmaceutical industry will also need careful consideration to identify any restrictions placed on approaches to study design by the regulatory bodies. Further, we are also aware of a potential tension between trialists who may believe their trials examine unique hypotheses (e.g. using modified interventions, or different populations from those used previously) and thus be reticent about the approach compared to the meta-analyst who takes a more holistic approach to evaluation. Trials which may be underway, but which have not yet reported their results, would need building into the simulation modelling, if they exist. The dilemmas facing trialists, when results of other trials become available during the duration of their trial, have been described previously [45]. Further, retrospective assessment of the use of such data via a cumulative meta-analysis to design and monitor a further trial has been proposed, [46] and is consistent with the evidence-based principles of the framework considered here. Additionally, issues relating to the monitoring of (individual) future trials would need careful consideration. Currently, we encourage researchers powering their studies using traditional methods to carry out a simultaneous assessment of how such studies would impact on existing meta-analyses so they are at least aware of the potential impact (or lack of) their study will have on the total evidence-base.

We would like to think in the future that the framework could be refined as relevant methodologies improve. For example, we welcome the day when IPD for all trials is made publicly available and reliance on published data could be removed from the framework. Additionally, evidence from observational studies may exist which could augment that available from the RCTs. While methodologies to synthesise both are in development [47] we believe they are some way off being recommended for routine use, but as approaches to adjust for biases develop [48] we hope this situation will change in the future. Indeed, we hope the publication of this paper will pave the way for a full scale pilot of the framework in a real clinical example. This would further identify the benefits and shortcomings of the approach (which may be addressable by further refinement and modification).

By considering equivalence limits for meta-analysis, emphasis is moved from statistical to clinical significance. However, often, several outcome measures (including those relating to adverse events) will be relevant to the decision on which treatment is superior. For this purpose, decision models can be built which include results from meta-analyses of multiple outcome measures [49,50]. Similarly, often, it will be necessary to consider *cost*-effectiveness of an intervention, which adds a further dimension of evidence which needs considering. Again, decision models are often developed in order to do this. In many instances, it will be appropriate to use parameter estimates derived from meta-analyses in such models [51] and as such they can be thought of as extended evidence synthesis models. Such models could be directly incorporated into the framework (Figure 1) alongside the IPD and MTC models.

A further step still would be to use a full decision theoretic approach to resource allocation and decision making [52]. Expected value of information (EVI) approaches to decision making are being developed [53,54]. Here, the potential payoffs of carrying out research in monetary terms are considered alongside the expense of carrying out the research. Although conceptually appealing, there are still difficult issues to resolve such as the need to estimate parameters in the model on which it is difficult to collect information. Our approach could be considered a less radical and (currently) more practical "halfway-house" between current practice and this ideal.

## Conclusion

While the collation of existing evidence as the basis for clinical practice is now routine, a more coherent and efficient approach to planning future RCTs to strengthen the evidence base needs to be developed. The framework presented is a proposal for how this situation can be improved.

## Competing interests

The authors declare that they have no competing interests.

## Authors' contributions

AS contributed to the i) conception and design; ii) analysis and interpretation of data; iii) drafting of the manuscript; iv) critical revision of the manuscript for important intellectual content; and v) statistical analysis. NC contributed to the i) conception and design; ii) drafting of the manuscript; and iii) critical revision of the manuscript for important intellectual content. DJ contributed to the i) conception and design; ii) drafting of the manuscript; and iii) critical revision of the manuscript for important intellectual content. All authors read and approved the final manuscript.

## Pre-publication history

The pre-publication history for this paper can be accessed here:

http://www.biomedcentral.com/1471-2288/9/29/prepub
